# Circ‐ILF2 in oral squamous cell carcinoma promotes cisplatin resistance and induces M2 polarization of macrophages

**DOI:** 10.1111/jcmm.17998

**Published:** 2023-10-20

**Authors:** Siyuan Wu, Xiaozhi Lv, Haigang Wei, Wuya Chen, Junming Zheng, Xia Li, Jing Song, Yilong Ai, Chen Zou

**Affiliations:** ^1^ Foshan Stomatological Hospital, School of Medicine Foshan University Foshan China; ^2^ Department of Oral and Maxillofacial Surgery, ZhuJiang Hospital Southern Medical University Guangzhou China

**Keywords:** circ‐ILF2, cisplatin resistance, M2 polarization of macrophages, oral squamous cell carcinoma

## Abstract

Cisplatin (CDDP) chemoresistance is one of the predominant factors in oral squamous cell carcinoma (OSCC) treatment failure. Uncovering the mechanisms underlying CDDP resistance is of great importance in OSCC therapy. Circular RNAs (circRNAs) are a newly discovered class of noncoding RNAs, which are reported to participate in the progression of various diseases, including cancer. However, the function of circRNAs in CDDP resistance in OSCC remains unclear. Quantitative reverse transcription PCR was used to search for different circRNAs between OSCC cell lines and CDDP‐resistant cell lines. The results showed that circ‐ILF2 expression was higher in CDDP‐resistant OSCC cell lines. The stability of circ‐ILF2 was also confirmed using RNase R and actinomycin D assays. Functional experiments, including cytotoxicity, apoptosis and growth rate assays, showed that upregulation of circ‐ILF2 contributes to CDDP resistance. Luciferase reporter‐gene, RNA pull‐down and quantitative real‐time PCR (RT‐qPCR) assays showed that circ‐ILF2 functions as a microRNA sponge for miR‐1252. Luciferase reporter assays, RNA pull‐down, RT‐qPCR and Western blotting showed that miR‐1252 directly targeted and regulated the expression of KLF8. Circ‐ILF2 plays an important role in CDDP resistance in OSCC. Circ‐ILF2 exerts its function through the miR‐1252/KLF8 pathway. In addition, tumour‐associated macrophages (TAM) play important roles in cancer progressions, our results showed that circ‐ILF2 in OSCC cells induced the M2 polarization of macrophages which provided new thoughts on immunotherapy. Our results suggest that circ‐ILF2 may represent a potential therapeutic target in CDDP‐resistant OSCC.

## 1 INTRODUCTION

Oral squamous cell carcinoma (OSCC) is one of the most common cancers and represents a subset of head and neck squamous cell carcinomas (HNSCCs).^1^, ^2^, ^3^ OSCC is a significant global health issue.^4^ Standard therapies for OSCC include surgery, chemotherapy and radiation.^5^, ^6^ Cisplatin (CDDP) is a first‐line drug for OSCC therapy. However, a major obstacle in OSCC therapy is that both innate and acquired CDDP resistance occur frequently in patients. The mortality rate of OSCC patients remains high.^7^, ^8^ Therefore, exploring the mechanisms underlying CDDP‐resistant OSCC is of great value.

Circular RNAs (circRNAs) are a newly discovered class of noncoding RNAs (ncRNAs) which have a closed loop structure.^9^ circRNAs are generated from the backsplicing of pre‐mRNA and are very stable and conserved because of their closed loop structure.^10^, ^11^ CircRNAs have been reported to play important roles in the progression of multiple diseases, including cancer. circRNAs exert their functions mainly by acting as competitive endogenous RNAs to sponge micro RNAs (miRNAs) and regulate the expression of miRNA target genes. miRNAs regulate target genes by binding to 3′‐UTR regions and exert their function through the target gene.^12^, ^13^ It has been reported that various circRNAs are widely expressed in OSCC patients. Ascending evidence reveals that the abnormal expression of circRNAs is related to the development and prognosis of OSCC. The circEPSTI1/miR‐942‐5p/LTBP2 axis regulates the progression of OSCC in the background of OSF via EMT and the PI3K/Akt/mTOR pathway.^14^ circIGHG‐induced epithelial‐to‐mesenchymal transition promotes oral squamous cell carcinoma progression via miR‐142‐5p/IGF2BP3 Signalling.^15^ Circle RNA hsa_circRNA_100290 serves as a ceRNA for miR‐378a to regulate oral squamous cell carcinoma cells growth via Glucose transporter‐1 (GLUT1) and glycolysis.^16^ Microarray profile of circular RNAs identifies hsa_circRNA_102459 and hsa_circRNA_043621 as important regulators in oral squamous cell carcinoma.^17^ Up to now, various circRNA‐miRNA‐mRNA networks have been found to be closely connected with the OSCC process.^15^, ^18^, ^19^

It has been reported that tumour‐associated macrophages (TAMs) play important roles in cancer progressions, including cancer cell proliferation, extracellular matrix remodelling, angiogenesis, metastasis and immunosuppression, as well as chemoresistance.^20^, ^21^ Two major states have been identified in macrophages, the classically activated type 1 (M1) and the alternatively activated type 2 (M2). M1 macrophages are characterized by the secretion of pro‐inflammatory cytokines like IL‐1β, TNF‐α, IL‐6 and IL‐12. They are also characterized by the expression of CD86, MCP1 and CD16. M2 characterized by anti‐inflammatory cytokines like IL‐10 and TGF‐β. They are also characterized by the expression of CD206, Arg‐1.^22^ TAMs constitute a main population of the OSCC microenvironment, with bipolar roles in cancer progression depending on their activation status (M1 vs. M2). The predominance of M1 or M2 phenotype in OSCC patients has been significantly associated with the disease outcome.^23^ M1‐like tumour‐associated macrophages activated by exosome‐transferred THBS1 promote malignant migration in oral squamous cell carcinoma.^24^ M1‐like tumour‐associated macrophages cascade a mesenchymal/stem‐like phenotype of oral squamous cell carcinoma via the IL6/Stat3/THBS1 feedback loop.^25^ RACK1 promotes cancer progression by increasing the M2/M1 macrophage ratio via the NF‐κB pathway in oral squamous cell carcinoma.^26^ Tumour‐associated macrophages promote oral cancer progression through activation of the Axl signalling pathway.^27^

In our study, we found that circ‐ILF2 (hsa_circ_001428) was upregulated in CDDP‐resistant OSCC cells compared to that in CDDP‐sensitive cells. Further research showed that circ‐ILF2 acts as a microRNA sponge for miR‐1252 and enhances CDDP resistance in OSCC through the regulation of KLF8. In addition, tumour‐associated macrophages (TAM) play important roles in cancer progressions, our results showed that circ‐ILF2 in OSCC cells induced the M2 polarization of macrophages which provided new thoughts on immunotherapy. Our results suggest that circ‐ILF2 may represent a potential therapeutic target in CDDP‐resistant OSCC. This study provides new insights into CDDP‐resistance research.

## 2 MATERIALS AND METHODS

### 2.1 Cell lines and culture

The human OSCC cell lines HSC3 and SCC25 were obtained from the Chinese Academy of Sciences Cell Bank (Shanghai, China). All cell lines were cultured at 37°C under 5% CO_2_ according to the supplier's instructions. CDDP resistance was induced by culturing SCC25 and HSC3 cells in 0.5 μM, 1 μM, 2 μM, 3 μM, 4 μM and 5 μM for 20 days respectively.

### 2.2 RNA isolation and quantitative real‐time PCR (RT‐qPCR)

Total RNA was extracted using TRIzol reagent (Invitrogen). A NanoDropND‐2000 spectrophotometer (Thermo Fisher Scientific) was used to determine the quality and quantity of RNA. DNase I was used to digest genomic DNA. A RiboMinus kit (Life Technologies, Thermo Fisher Scientific) was used to remove ribosomal RNAs. The treated RNA (0.5 μg) was reverse‐transcribed using Prime Script RT Master Mix (Takara, Japan). A New Poly(A) Tailing Kit (Thermo Fisher Scientific) was used for microRNA (miRNA) reverse transcription. The PrimeScript RT Master Mix Kit (Takara, RR036A, Japan) was used for cDNA synthesis. RT‐qPCR was performed using Universal SYBR Green Master Mix (Roche, Shanghai, China). The 2^−△△CT^ method was used for relative gene expression analysis. The primers are shown as followings:

**Table:** 

Gene	Forward primer	Reverse primer
SOX6	GGATGCAATGACCCAGGATTT	TGAATGGTACTGACAAGTGTTGG
E2F4	CACCACCAAGTTCGTGTCCC	GCGTACAGCTAGGGTGTCA
TCF4	CAAGCACTGCCGACTACAATA	CCAGGCTGATTCATCCCACTG
KLF6	GGCAACAGACCTGCCTAGAG	CTCCCGAGCCAGAATGATTTT
E1F1	ACCATACCAGATGTAGAGGACTC	GTTACCACTATCACCAGGGGG
KLF8	CCCAAGTGGAACCAGTTGACC	GACGTGGACACCACAAGGG
AKT3	TGTGGATTTACCTTATCCCCTCA	GTTTGGCTTTGGTCGTTCTGT
FGF2	AGAAGAGCGACCCTCACATCA	CGGTTAGCACACACTCCTTTG
HLF	CCACCTTTATCCCGCCTCC	TTTACTAAATGCGTCTTCGTGGT
KIF14	CCTCACCCACAGTAGCCGA	AAGTGCCAATCTACCTACAGGA
IL‐1β	ATGATGGCTTATTACAGTGGCAA	GTCGGAGATTCGTAGCTGGA
TNF‐α	CCTCTCTCTAATCAGCCCTCTG	GAGGACCTGGGAGTAGATGAG
CD16	CCTCCTGTCTAGTCGGTTTGG	TCGAGCACCCTGTACCATTGA
MCP1	CAGCCAGATGCAATCAATGCC	TGGAATCCTGAACCCACTTCT
TGF‐β	GGCCAGATCCTGTCCAAGC	GTGGGTTTCCACCATTAGCAC
Arg‐1	GTGGAAACTTGCATGGACAAC	AATCCTGGCACATCGGGAATC
IL‐10	GACTTTAAGGGTTACCTGGGTTG	TCACATGCGCCTTGATGTCTG
CD206	TCCGGGTGCTGTTCTCCTA	CCAGTCTGTTTTTGATGGCACT
GAPDH	GGAGCGAGATCCCTCCAAAAT	GGCTGTTGTCATACTTCTCATGG

### 2.3 RNase R treatment

1 mg of total RNA was treated with RNase R for 20 min at 37°C according the manual of the manuscript (RNR07250, Lucigen). Then, qRT‐PCR was performed to detect the expression of circRNA and linear RNA. 1% agarose gel was used for DNA electrophoresis.

### 2.4 Actinomycin D assay

OSCC cells were cultured in a 24 well plate. OSCC cells were treated with 2.5 mg/L actinomycin D (Sigma‐Aldrich, USA). Cells were collected and RNA was extracted at 0, 4, 8, 12 and 24 h. The stability and expression levels of circRNA and mRNA were examined by qRT‐PCR.

### 2.5 Cell viability assay

OSCC cell viability was detected by Cell counting kit‐8 (CCK‐8) assay. OSCC cells were seeded at a density of 6000 cells per well. 10 μL CCK8 was added per well. The plate was incubated 4 h at 37°C before detection.

### 2.6 Dual luciferase reporter assays

The sequence contains of circ‐ILF2 and KLF8 3′UTR were cloned into pGL3 luciferase promoter plasmid (Promega Corporation). Mutations of the microRNA binding sites in circRNA and KLF8 were synthesized using the QuickChange Mutagenesis Kit (Stratagene). Constructed pGL3 plasmids were cotransfected with microRNA mimics, miR‐1252 or miR‐1252 inhibitors in OSCC cells using Lipofectamine 3000 (Invitrogen). The activities of luciferase reporters were detected 48 h later.

### 2.7 RNA pull‐down assay

RNA pull‐down assays were carried out as previously reported methods.^15^ Biotin‐labelled circ‐ILF2 was designed and synthesized by RiboBio (Guangzhou, China). Biotin‐labelled miR‐1252 was designed and synthesized by GenePharma (Shanghai, China). OSCC cell lysate was cultured with streptavidin‐coated beads. The directly the binding complex was pulled down and detected by qRT‐PCR.

### 2.8 Isolation of nuclear and cytoplasmic fractions

PARIS™ Kit (Ambion) was applied to isolate nuclear and cytoplasmic RNA. qRT‐PCR was used to detect the nuclear and cytoplasmic RNA, respectively. U6 was used as the nucleus control, and GAPDH was used for cytoplasm control.

### 2.9 Cell transfections

Lentivirus for circ‐ILF2, miR‐1252, miR‐1252 inhibitor and shKLF8 were obtained from GenePharma (Shanghai, China). Puromycin (Solarbio, China) was used to establish stable cell lines transfected with the indicated lentivirus.

### 2.10 Western blot

Protein was extracted from OSCC cells with a protein extraction kit (Key Gene, KGP9100) with Protease Inhibitor Cocktail (Roche, Basel, Switzerland). The concentration of protein was detected by BCA kit (Thermo Fisher Scientific). 30 μg proteins were loaded for detection. The protein was separated by 8%, 12.5% or 15% gels and transferred onto PVDF membrane (Merck Millipore). The membrane was incubated with the indicated antibodies overnight at 4°C after 5% nonfat milk blockage. Then, the membrane was washed three times for 10 min in PBST and cultured with secondary antibodies for 2 h. ECL detection system (Thermo Fisher Scientific) was used to detect the protein level. β‐actin was used as the loading control.

### 2.11 THP‐1 cell differentiation

THP‐1 cells were treated with phorBOL‐12‐myriSTATE‐13‐acetate (PMA, 20 ng/mL). THP‐1 monocytes were induced to a macrophage‐like (M0) phenotype in sigma‐Aldrich's 5% FBS‐RPMI medium for 48 h, and then cultured in 10% FBS‐RPMI control medium for 24 h. Cells were collected and inoculated on 6‐well plates and cocultured with conditioned medium from SCC25R and HSC3R.

### 2.12 Enzyme‐linked immunosorbent assay (ELISA)

The protein levels of IL‐1β (Abcam), and TNF‐α (R&D Systems) in the supernatants of 1.5 × 10^6^ macrophages were measured using a human ELISA kit according to the manufacturer's protocols.

### 2.13 Flow cytometry

The THP‐1 derived macrophages were treated with different conditioned medium for 48 h, and then, macrophages were treated with 0.25% trypsin, centrifuged at 500 *g* for 3 min. Macrophages were resuspended with staining buffer, and then incubated with M1 macrophages biomarker antihuman CD86 and M2 macrophages biomarker antihuman CD206 for 35 min. Macrophages were detected using a FACSCalibur Flow Cytometer (BD) and analysed using FlowJo software (Tree Star Inc).

### 2.14 Statistical analysis

All data were shown as mean ± SD. GraphPad Prism 6 (GraphPad Software Inc.) was used for statistical analysis. The difference between the two groups was identified using the unpaired two‐tailed Student's *t*‐test. Multiple comparisons were identified by two‐way anova. *p* < 0.05 was considered statistically significant. *p < 0.05, **p < 0.01, ***p < 0.001.

## 3 RESULTS

### 3.1 Circ‐ILF2 is overexpressed in CDDP‐resistant OSCC cells

**FIGURE 1:**
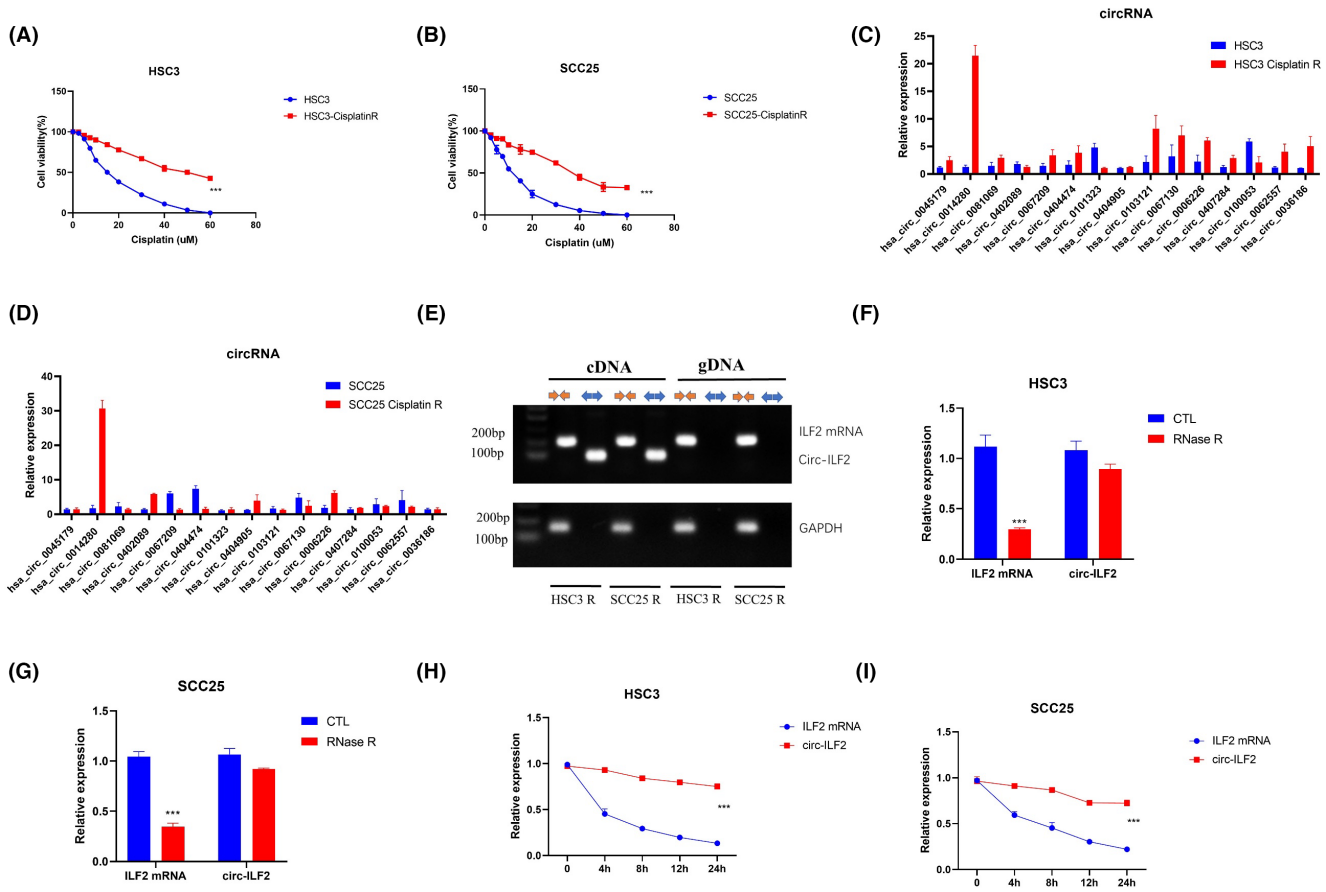
Circ‐ILF2 upregulation in cisplatin (CDDP)‐resistant oral squamous cell carcinoma (OSCC) cells. (A) CCK8 assays were used to detect cell viability of HSC3 and CDDP‐resistant HSC3 (HSC3 R) cells after treatment with CDDP. (B) CCK8 assays were used to detect cell viability of SCC25 and CDDP resistant SCC25 (SCC25 R) cells after treatment with CDDP. (C) RT‐qPCR‐based identification of circular RNAs (circRNAs) in HSC3 and HSC3 R cells. (D) RT‐qPCR‐based identification of circRNAs in SCC25 and SCC25 R cells. (E) RT‐qPCR‐based detection of circ‐ILF2 and linear ILF2 in cDNA or genomic DNA (gDNA). (F) RNase R assay was performed in HSC3 to detect circ‐ILF2 and *ILF2* mRNA. (G) RNase R assay was performed in SCC25 cells to detect circ‐ILF2 and *ILF2* mRNA. (H) Actinomycin D assay was performed in HSC3 cells. (I) Actinomycin D assay was performed in SCC25 cells. *p < 0.05, **p < 0.01, ***p < 0.001.

Increasing research has shown that circRNAs are involved in various cancer processes.^28^, ^29^ To investigate the function of circRNA in CDDP‐resistant OSCC, we established two CDDP‐resistant OSCC cell lines, HSC3 R and SCC25 R, by culturing OSCC cells with increasing concentrations of CDDP for 4 months. We detected CDDP‐resistant OSCC cell lines using CCK8 assays. The results indicated that CDDP‐resistant HSC3 (HSC3 R) and SCC25 (SCC25 R) cells had greater cell viability than the HSC3 and SCC25 parental cell lines when treated with different concentrations of CDDP (Figure 1A,B). As circRNAs have been reported to play important roles in OSCC progression,^19^, ^30^, ^31^ we performed RT‐qPCR to detect circRNA differences in OSCC CDDP‐resistant cells and parental cells (Figure 1C, D). RT‐qPCR results showed that circ‐ILF2 (hsa_circrna_014280) was significantly upregulated in HSC3 R cells (Figure 1C) and in SCC25 R cells (Figure 1D). To confirm the presence of circ‐ILF2, we designed specific divergent and convergent primers to amplify *ILF2* linear RNA and circ‐ILF2. PCR results showed that circ‐ILF2 could only be amplified using cDNA, whereas *ILF2* linear RNA could be amplified from both genomic DNA and cDNA (Figure 1E). We also performed RNase R and actinomycin D assays to confirm the stability of the circRNA. The results showed that circ‐ILF2 was more resistant to RNase R digestion than its linear isoform, further confirming the loop form of circ‐ILF2 (Figure 1F,G). Actinomycin D assays showed that the half‐life of circ‐ILF2 exceeded 24 h, whereas that of the linear RNA was <8 h (Figure 1H,I). Taken together, our results indicated that circ‐ILF2 has a circular form and is more stable than its linear isoform.

### 3.2 Circ‐ILF2 is critical for CDDP resistance

**FIGURE 2:**
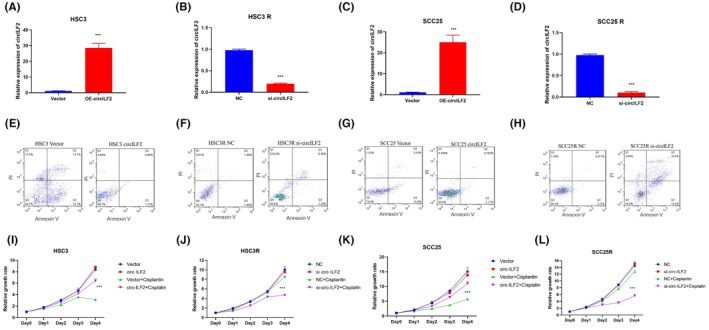
Circ‐ILF2 facilitates cisplatin (CDDP) resistance in oral squamous cell carcinoma (OSCC) in vitro. (A) RT‐qPCR was performed in HSC3 cells after transfection of circ‐ILF2. (B) RT‐qPCR was performed in HSC3 R cells with silencing of circ‐ILF2. NC means nonsense control. (C) RT‐qPCR was performed in SCC25 cells after transfection of circ‐ILF2. (D) RT‐qPCR was performed in SCC25 R cells with silencing of circ‐ILF2. (E) Flow cytometric analysis was used to detect apoptosis of HSC3 Vector and circ‐ILF2 cells treated with 1 μM CDDP for 48 h. (F) Flow cytometric analysis was used to detect apoptosis of HSC3 R, NC, and si‐circ‐ILF2 cells treated with 5 μM CDDP for 48 h. (G) Flow cytometric analysis was used to detect apoptosis of SCC25 Vector and circ‐ILF2 cells treated with 1 μM CDDP for 48 h. (H) Flow cytometric analysis was used to detect apoptosis of SCC25 R, NC, and circ‐ILF2 cells treated with 5 μM CDDP for 48 h. (I) CCK8 assay was used to detect the growth rate of HSC3 Vector and circ‐ILF2 cells treated with 1 μM CDDP. (J) CCK8 assay was used to detect the growth rate of HSC3 R, NC, and si‐circ‐ILF2 cells treated with 5 μM CDDP. (K) CCK8 assay was used to detect the growth rate of SCC25 Vector and circ‐ILF2 cells treated with 1 μM CDDP. (L) CCK8 assay was used to detect the growth rate of SCC25 R NC and circ‐ILF2 cells treated with 5 μM CDDP. *p < 0.05, **p < 0.01, ***p < 0.001.

To investigate the function of circ‐ILF2 in CDDP resistance, we designed circ‐ILF2 siRNA and circ‐ILF2‐overexpressing plasmids. RT‐qPCR was used to detect the effects of overexpression of HSC3 (Figure 2A) and SCC25 (Figure 2C). Silencing effects in HSC3 R (Figure 2B) and SCC25 R (Figure 2D) were also detected by q‐PCR.

We performed apoptosis and cell growth assays to examine the effects of circ‐ILF2 on CDDP resistance. As shown in Figure 2E,G, circ‐ILF2 overexpression significantly decreased CDDP‐induced apoptosis in HSC3 (Figure 2E) and SCC25 cells (Figure 2G). Silencing circ‐ILF2 significantly increased CDDP‐induced apoptosis (Figure 2F,H). Moreover, overexpression of circ‐ILF2 significantly weakened CDDP‐induced cell growth inhibitory effects in HSC3 (Figure 2I) and SCC25 cells (Figure 2K). Silencing of circ‐ILF2 led to remarkable cell growth inhibitory effects in HSC3 R (Figure 2J) and SCC25 R cells (Figure 2L). These results confirmed the specific role of circ‐ILF2 in the maintenance of CDDP resistance.

### 3.3 Circ‐ILF2 acts as an efficient microRNA sponge for miR‐1252

**FIGURE 3:**
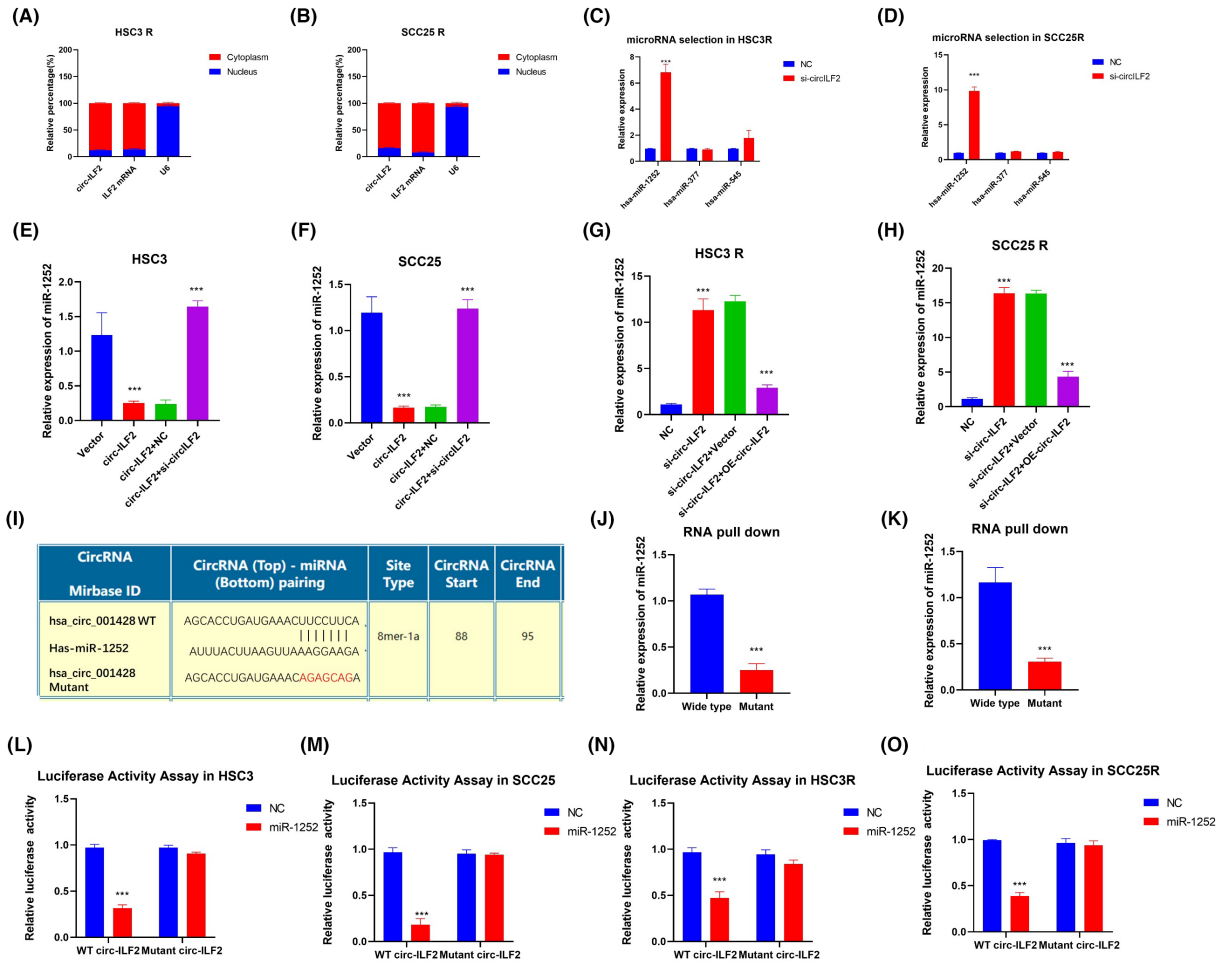
Circ‐ILF2 acts as microRNA sponge for miR‐1252. (A) Levels of circ‐ILF2 and *ILF2* mRNA in cytoplasmic and nuclear fractions of HSC3 R cells. (B) Levels of circ‐ILF2 and *ILF2* mRNA in cytoplasmic and nuclear fractions of SCC25 R cells. (C) RT‐qPCR was used to detect the predicted target microRNAs of circ‐ILF2 in HSC3 R cells. NC means nonsense control. (D) RT‐qPCR was used to detect the predicted target microRNAs of circ‐ILF2 in SCC25 R cells. (E) Levels of miR‐1252 were detected by RT‐qPCR with rescue experiments in HSC3 cells. (F) The level of miR‐1252 was detected by RT‐qPCR with rescue experiments in SCC25 cells. (G) The level of miR‐1252 was detected by RT‐qPCR with rescue experiments in HSC3 R cells. (H) The level of miR‐1252 was detected by RT‐qPCR with rescue experiments in SCC25 R cells. (I) Sequence of wild‐type circ‐ILF2 and circ‐ILF2 with mutations. (J) RNA pull‐down assay was performed with wild‐type and mutated circ‐ILF2 in HSC3 R cells. (K) RNA pull‐down assay was performed with wild‐type and mutated circ‐ILF2 in SCC25 R cells. (L) Luciferase activity of wild‐type or mutated circ‐ILF2 was detected in HSC3 cells after cotransfection with nonsense control or miR‐1252. (M) Luciferase activity of wild‐type or mutated circ‐ILF2 was detected in SCC25 cells after cotransfection with nonsense control or miR‐1252. (N) Luciferase activity of wild‐type or mutated circ‐ILF2 was detected in HSC3 R cells after cotransfection with nonsense control or miR‐1252. (O) Luciferase activity of wild‐type or mutated circ‐ILF2 in SCC25 R cells was detected after cotransfection with nonsense control or miR‐1252. *p < 0.05, **p < 0.01, ***p < 0.001.

It is well known that many circRNAs exert their function by acting as sponges for microRNAs in the cytoplasm. We performed nuclear and cytoplasmic extraction experiments and detected the expression of circ‐ILF2 by RT‐qPCR. The results showed that circ‐ILF2 mainly existed in the cytoplasm, indicating that circ‐ILF2 might act as a miRNA sponge (Figure 3A,B). CircInteractome (Circular RNA interactome https://circinteractome. nia.nih.gov/) was used to investigate possible microRNA response elements harboured by circ‐ILF2. CircInteractome analysis showed that there were three possible miRNA targets. RT‐qPCR was used to detect the influence of circ‐ILF2 on three possible miRNAs. The results showed that silencing of circ‐ILF2 in HSC3 R and SCC25 R cells significantly upregulated the expression of miR‐1252 without influencing miR‐377 and miR‐545. To confirm the regulation of circ‐ILF2 on miR‐1252, we performed rescue experiments using OSCC cells. The results showed that overexpression of circ‐ILF2 decreased the expression of miR‐1252, and silencing of circ‐ILF2‐abrogated circ‐ILF2‐induced miR‐1252 upregulation in HSC3 (Figure 3E) and SCC25 cells (Figure 3F). Silencing circ‐ILF2 significantly upregulated the expression of miR‐1252 in HSC3 R (Figure 3G) and SCC25 R (Figure 3H) cells, and re‐expression of circ‐ILF2 blocked the upregulation of miR‐1252 induced by circ‐ILF2 silencing. To confirm the direct interaction between circ‐ILF2, we mutated the binding sites on circ‐ILF2 (Figure 3I). RNA pull‐down assays were performed using biotin‐labelled circ‐ILF2 probes. As shown in Figure 3J,K, miR‐1252 could be pulled‐down by wild‐type circ‐ILF2, whereas mutation of the binding sites abrogated the direct interaction between miR‐1252 and circ‐ILF2. We constructed wild‐type or binding site‐mutated circ‐ILF2 pGL3 plasmids. Luciferase assay results showed that transfection with miR‐1252 significantly decreased the activity of the wild‐type circ‐ILF2 reporter rather than the mutated circ‐ILF2 reporter, suggesting that miR‐1252 and circ‐ILF2 interacted directly (Figure 3L–O). Taken together, these results suggested that circ‐ILF2 functions as a sponge for miR‐1252.

### 3.4 *KLF8* is a direct target gene of miR‐1252 and is regulated by circ‐ILF2

**FIGURE 4:**
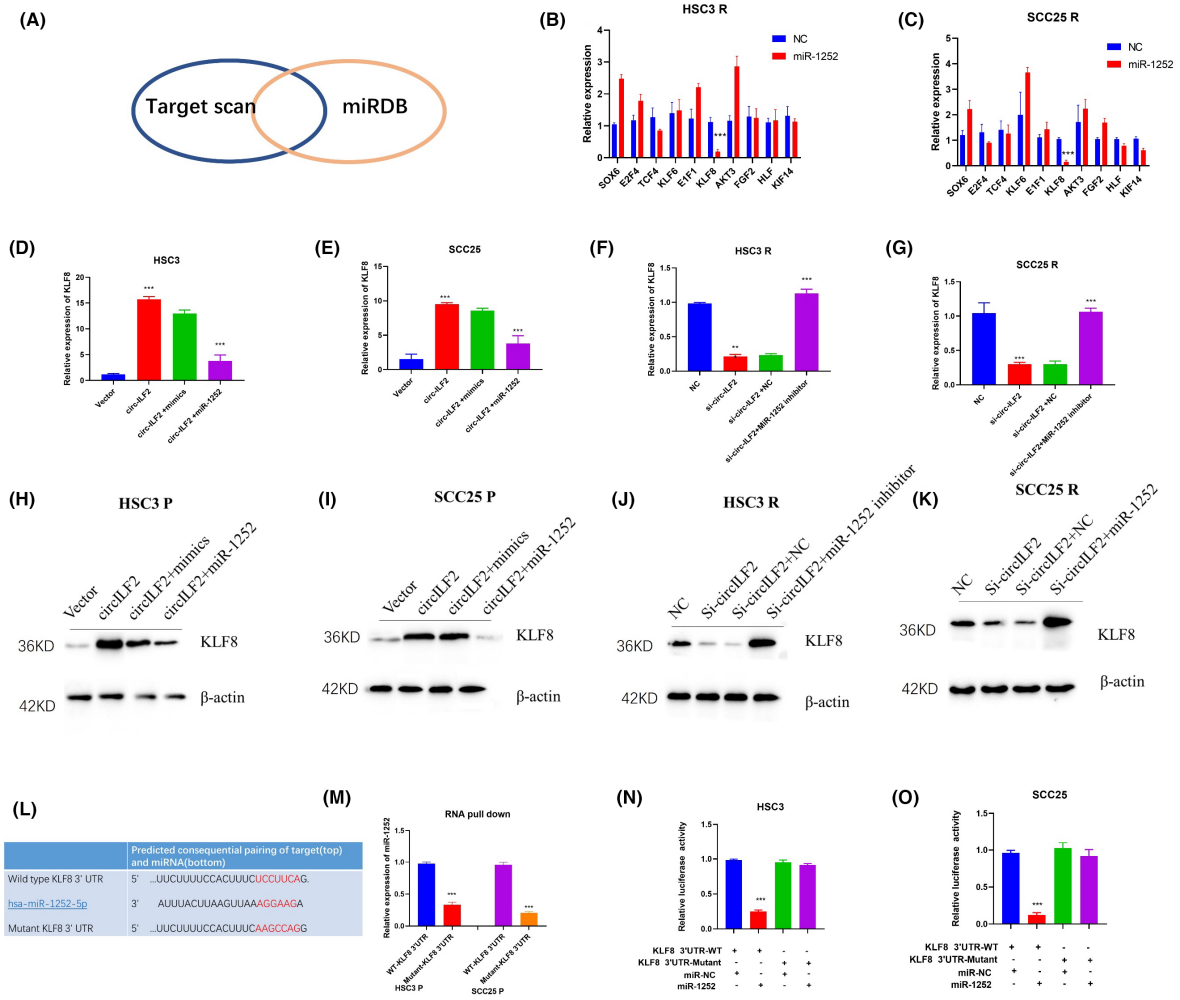
*KLF8* is a direct target of miR‐1252. (A) Overlapping of the target genes of circ‐ILF2 based on Targetscan and miRDB. (B) RT‐qPCR was used to detect the expression of predicted genes after transfection of nonsense (NC) or miR‐1252 in HSC3 R cells. (C) RT‐qPCR was used to detect the expression of predicted genes after transfection of nonsense (NC) or miR‐1252 in SCC25 R cells. (D,E) RT‐qPCR was used to detect the expression of *KLF8* after transfection of circ‐ILF2 or miR‐1252 in HSC3 and SCC25 cells. (F,G) RT‐qPCR was used to detect the expression of *KLF8* after transfection of si‐circ‐ILF2 or miR‐1252 inhibitor in HSC3 R and SCC25 R cells. (H,I) Western blotting was used to detect the expression of KLF8 after transfection of circ‐ILF2 or miR‐1252 in HSC3 and SCC25 cells. (J,K) Western blotting was used to detect the expression of KLF8 with transfection of si‐circ‐ILF2 or miR‐1252 inhibitor in HSC3 R and SCC25 R cells. (L) Mutation of the binding site on *KLF8* 3′‐UTR. (M) RNA pull‐down assay was performed with wild‐type and mutated *KLF8* in HSC3 P and SCC25 P cells. (N) Luciferase activity of wild‐type or mutated *KLF8* 3′‐UTR in HSC3 cells was detected after cotransfection with nonsense control or miR‐1252 in HSC3 cells. (O) Luciferase activity of wild‐type or mutated *KLF8* 3′‐UTR in HSC3 cells was detected after cotransfection with nonsense control or miR‐1252 in SCC25 cells. *p < 0.05, **p < 0.01, ***p < 0.001.

TargetScan and miRDB databases were used to predict potential targets of miR‐1252. We selected 10 genes listed in the miRNA target gene databases. Expression of the target genes was detected by RT‐qPCR after transfection of miR‐1252 into CDDP‐resistant OSCC cells (Figure 4B, C). The results showed that silencing of miR‐1252 significantly decreased the mRNA levels of Krüppel‐like factor 8 (*KLF8*) in both HSC3 R (Figure 4B) and SCC25 R (Figure 4C) cells. KLF8 regulates the transcription of genes involved in diverse biological processes, including proliferation, drug resistance and inflammation. KLF8 is widely expressed in different tissues and has been confirmed to be aberrantly expressed in various cancers. Previous studies have reported that KLF8 plays an important role in cancer chemoresistance. We performed RT‐qPCR and Western blotting to confirm KLF8 expression. The results showed that KLF8 levels were upregulated by circ‐ILF2 overexpression and downregulated by miR‐1252 transfection in HSC3 and SCC25 cells (Figure 4D,E,H,I). KLF8 expression was significantly reduced by circ‐ILF2 siRNA, whereas this effect was reversed by transfection of miR‐1252 inhibitor in HSC3 R and SCC25 R cells (Figure 4F,G,J,K). To search for the binding site between miR‐1252 and KLF8 mRNA, RNA pull‐down and luciferase activity assays were performed. As shown in Figure 4L,M, miR‐1252 could be pulled‐down by the wild‐type *KLF8* 3′‐UTR, whereas mutation of the binding sites abrogated the direct interaction between miR‐1252 and *KLF8*. We also inserted the wild‐type and mutated sequences of KLF8 3′‐UTR, which contained the predicted binding site, into pGL3 luciferase reporter‐gene plasmids (Figure 4L). Luciferase activity assay results showed that the luciferase activity of wild‐type *KLF8* 3′‐UTR plasmids was significantly reduced after transfection with miR‐1252, whereas that of plasmids containing mutant *KLF8* 3′‐UTR was not influenced by miR‐1252 (Figure 4N,O). In conclusion, we found that KLF8 expression is regulated by circ‐ILF2/miR‐1252.

### 3.5 miR‐1252/KLF8 response to effects of circ‐ILF2 on CDDP‐resistant OSCC cells

**FIGURE 5:**
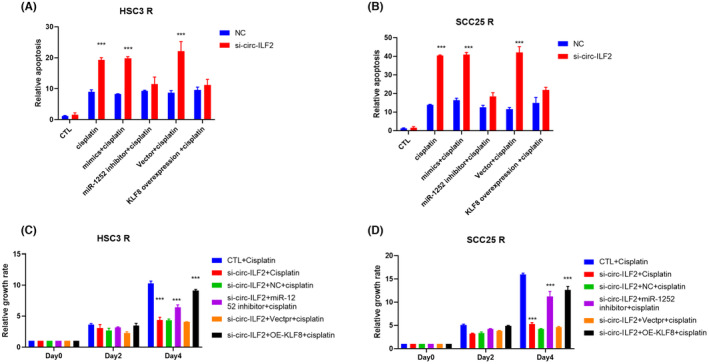
Circ‐ILF2/miR‐1252/KLF8 contributes to cisplatin (CDDP)‐resistant oral squamous cell carcinoma (OSCC). (A) Apoptosis assays were performed in HSC3 R NC/si‐circ‐ILF2 cells after transfection of miR‐1252 inhibitor or KLF8 overexpression plasmids with treatment of 5 μM cisplatin for 48 h. (B) Apoptosis assays were performed in SCC25 R NC/si‐circ‐ILF2 cells after transfection of miR‐1252 inhibitor or KLF8 overexpression plasmids with treatment of 5 μM cisplatin for 48 h. (C) Growth rates were detected in HSC3 R NC/si‐circ‐ILF2 cells after transfection of miR‐1252 inhibitor or KLF8 overexpression plasmids with treatment of 5 μM cisplatin. (D) Growth rates were detected in SCC25 R NC/si‐circ‐ILF2 cells after transfection of miR‐1252 inhibitor or KLF8 overexpression plasmids with treatment of 5 μM cisplatin. *p < 0.05, **p < 0.01, ***p < 0.001.

To examine the effects of circ‐ILF2/miR‐1252/KLF8 on CDDP‐resistant OSCC cells, HSC3 R and SCC25 R cells were transfected with miR‐1252 inhibitor or KLF8 expression plasmids after circ‐ILF2 silencing. Flow cytometry was used to detect apoptosis after CDDP treatment in HSC3 R and SCC25 R cells, and the results showed that the increase in apoptosis caused by circ‐ILF2 silencing was inhibited by miR‐1252 inhibitor or KLF8 overexpression in HSC3 R (Figure 5A) cells. Similar results were obtained for SCC25 R (Figure 5B). The influence of circ‐ILF2/miR‐1252/KLF8 on OSCC growth was assessed using CCK8 assays. The results of the CCK8 assays showed that cell growth inhibition induced by circ‐ILF2 silencing was restored by miR‐1252 inhibitor or KLF8 overexpression in HSC3 R (Figure 5C) cells. Similar results were obtained for SCC25 R (Figure 5D). In summary, miR‐1252/KLF8 is responsible for circ‐ILF2‐induced CDDP resistance in OSCC cells.

### 3.6 Circ‐ILF2 in OSCC induced M2 polarization of macrophages

**FIGURE 6:**
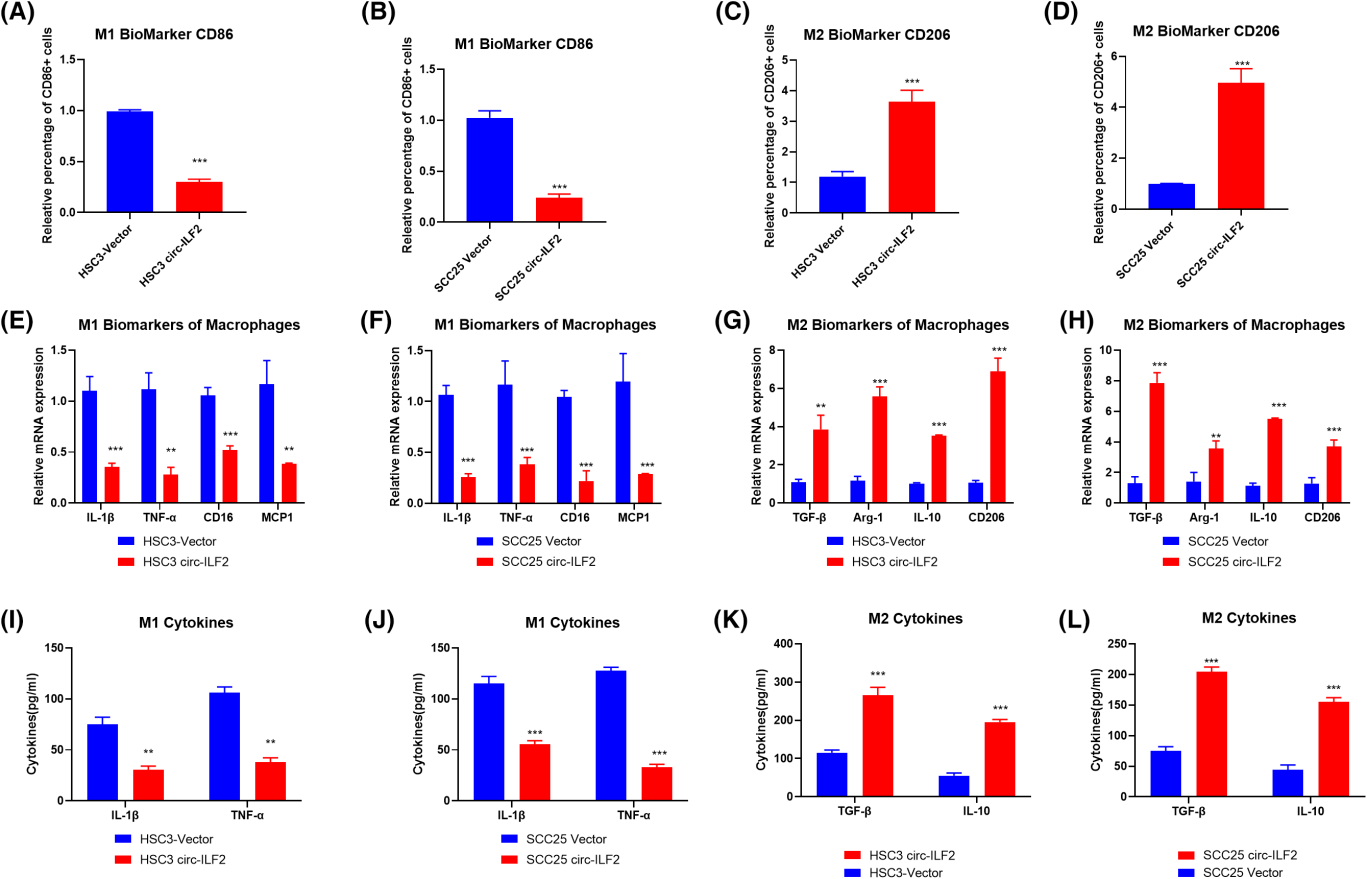
Circ‐ILF2 in OSCC induces M2 polarization of macrophages. (A,B) Flow cytometry was used to detect the surface expression of CD86 in macrophages cocultured with HSC3 (A) and SCC25 (B) for 48 h. (C,D) Flow cytometry was used to detect the surface expression CD206 in macrophages cocultured with HSC3 (C) and SCC25 (D) for 48 h. (E,F) qRT‐PCR was used to detect the expression of M1 markers (TNF‐α, IL‐1β, MCP‐1 and CD16) in macrophages cocultured with HSC3 (E) and SCC25 (F). (G,H) qRT‐PCR was used to detect the expression of M2 markers (Arg‐1, CD206, TGF‐β, IL‐10) in macrophages cocultured with HSC3 (G) and SCC25 (H). (I,J) ELISA was used to detect the expression of M1 inflammatory cytokines cocultured with HSC3 (I) and SCC25 (J). (K,L) ELISA was used to detect the expression of M2 inflammatory cytokines cocultured with HSC3 (I) and SCC25 (J). *p < 0.05, **p < 0.01, ***p < 0.001.

It has been reported that tumour‐associated macrophages (TAM) play important roles in cancer progressions. Two major states have been identified in macrophages, the classically activated type 1 (M1) and the alternatively activated type 2 (M2). We investigated the function of circ‐ILF2 in macrophages polarization. Therefore, in our study, the OSCC cells that interfered with circ‐ILF2 were cocultured with PMA‐induced THP‐1 cells for 48 h, and macrophage polarization markers were detected. The results showed that overexpression of circ‐ILF2 in HSC3 (Figure 6A,C) and SCC25 (Figure 6B,D) promoted the M2 polarization of macrophages. Similar results were obtained through using the detection of macrophage polarization‐related markers and inflammatory cytokines (Figure 6E–L).

## 4 DISCUSSION

CDDP is one of the most widely used drugs in OSCC cancer therapy.^32^, ^33^ Chemotherapy failure is an important cause of poor prognosis in OSCC patients. Uncovering the mechanisms underlying CDDP chemoresistance may provide new insights into OSCC treatment. With the development of second‐generation DNA sequencing technologies, circRNAs have been found to be abundantly expressed. CircRNAs are reported to be important in the progression of various cancers including colorectal cancer, bladder cancer prostate cancer and OSCC.^34^, ^35^, ^36^, ^37^ We performed qRT‐PCR in cisplatin‐resistant and parental OSCC cells to detect differences in circRNA expression. Circ‐ILF2 was chosen for further study because its expression was significantly upregulated in CDDP‐resistant OSCC cells compared to parental cells (Figure 1). RNase R and actinomycin D assays were used to confirm the stability of circRNAs in OSCC cells. Investigations of the function of circRNAs in chemoresistance remain very rare. In this study, we focused on the function and mechanism of circ‐ILF2 in CDDP‐resistant OSCC.

To explore the function of circ‐ILF2 in CDDP‐resistant OSCC, we obtained two CDDP‐resistant OSCC cell lines. The results showed that circ‐ILF2 was overexpressed in CDDP‐resistant HSC3 and SCC25 cells. RNase R and actinomycin D assays confirmed the high stability of circ‐ILF2 (Figure 1). Overexpression of circ‐ILF2 decreased CDDP‐induced apoptosis and inhibition effects on proliferation. Silence of circ‐ILF2 enhanced CDDP‐induced apoptosis and inhibition effects on proliferation (Figure 2).

circRNAs have been reported to function as miRNA sponges in the cytoplasm and thereby competitively regulate miRNA targets.^38^, ^39^ From our results, RT‐qPCR data of cytoplasmic and nuclear RNAs revealed that circ‐ILF2 mainly existed in the cytoplasm, indicating that circ‐ILF2 might act as a microRNA sponge and regulate miRNA targets in CDDP‐resistant OSCC. RT‐qPCR experiments were used to validate bioinformatically predicted downstream targeted microRNAs in HSC3 R and SCC25 R cells with circ‐ILF2 silencing. Results showed that silencing circ‐ILF2 significantly upregulated the expression of miR‐1252 without influencing other miRNAs. Rescue experiments confirmed that circ‐ILF2 specifically regulated the expression of miR‐1252. Furthermore, RNA pull‐down and luciferase activity experiments revealed that circ‐ILF2 directly bound to miR‐1252 (Figure 3).

Previous studies have shown that miR‐1252 participates in the progression of several cancers, including lung adenocarcinoma, pancreatic cancer and ovarian cancer. MiR‐1252 mainly exerts its function by acting as a downstream target of competitive endogenous RNA (ceRNAs).^40^, ^41^, ^42^ However, the target gene of miR‐1252 in OSCC has not yet been identified. In our study, we used TargetScan and miRDB databases to predict the target gene of miR‐1252. We performed RT‐qPCR and Western blotting to confirm the specific regulation of miR‐1252 on KLF8 expression.

KLF Transcription Factor 8 (KLF8) belongs to the Sp/KLF transcription factor family. KLF8 regulates the transcription of genes involved in diverse biological processes, such as proliferation, inflammation, migration and invasion.^43^ KLF8 is widely expressed in various tissues, and abnormal expression of KLF8 is manifested in several cancers, including lung, gastric and ovarian cancers.^44^, ^45^ Previous studies have reported that KLF8 plays important roles in DNA repair and apoptosis resistance.^43^ In our study, we found that KLF8 is a downstream target of miR‐1252 and that miR‐1252 is responsible for circ‐ILF2 induced KLF8 upregulation. In this study, we identified a novel regulation of KLF8 by the circ‐ILF2/miR‐1252 axis. Functional experiments confirmed that miR‐1252 inhibitor and KLF8 overexpression rescued circ‐ILF2 silencing‐induced apoptosis and growth repression following CDDP treatment (Figure 5). Our results revealed that KLF8 was a direct target of miR‐1252, and circ‐ILF2 increased KLF8 expression by weakening the inhibitory effects of miR‐1252 on KLF8 in OSCC.

Solid tumours comprise not only cancer cells but also immune cells and stromal cells such as adipocytes, fibroblasts and haematopoietic cells. Among the tumour infiltrated immune cells, macrophages are reported to be the most abundant, called tumour associated macrophages (TAMs). In our research, the results showed that circ‐ILF2 in OSCC cells could also promote the M2 polarization of macrophages. The mechanism of how circ‐ILF2 regulate M2 polarization of macrophages needs more investigation. What is more, a better understanding of function of M2 polarization on cisplatin resistance is also needed. In the future researches, we would focus on the roles of circRNAs in macrophages polarization. We intend to find out how circRNAs regulate the function of macrophages and how macrophages influence the cancer progressions.

## 5 CONCLUSIONS

In summary, our study identified that circ‐ILF2 is upregulated in CDDP‐resistant OSCC cells. circ‐ILF2 acts as a microRNA sponge to block the suppressive effect of miR‐1252 on KLF8 expression and contributes to CDDP‐resistant OSCC. In addition, our results showed that circ‐ILF2 in OSCC cells induced the M2 polarization of macrophages which provided new thoughts on immunotherapy. Our results suggest that circ‐ILF2 may represent a potential therapeutic target in CDDP‐resistant OSCC. These results provide novel insights into the mechanism of CDDP resistance.

## AUTHOR CONTRIBUTIONS

**Siyuan Wu:** Investigation (equal); methodology (equal); project administration (equal); writing – original draft (equal). **Xiao‐zhi Lv:** Investigation (supporting); methodology (equal); project administration (equal); writing – original draft (equal). **Haigang Wei:** Data curation (equal); validation (equal); visualization (equal); writing – original draft (equal). **Wuya CHEN:** Conceptualization (equal); software (equal); supervision (equal). **Junming ZHENG:** Data curation (equal); formal analysis (equal); supervision (equal). **Xia Li:** Supervision (equal); validation (equal); visualization (equal). **Jing Song:** Conceptualization (equal); data curation (equal); supervision (equal). **Yilong Ai:** Funding acquisition (equal); methodology (equal); supervision (equal). **Chen Zou:** Funding acquisition (equal); project administration (equal); supervision (equal); writing – original draft (equal).

## FUNDING INFORMATION

This work was supported by the Guangdong Basic and Applied Basic Research Foundation (2022A1515140098).

## CONFLICT OF INTEREST STATEMENT

The authors confirm that there are no conflicts of interest.

## ETHICS STATEMENT

Our study did not require an ethical board approval because it did not contain human or animal trials.

## Data Availability

The data of this study are available from the corresponding author upon reasonable request
